# Blockchain-driven vocational higher education framework for industry-academia-research collaboration within digital public health context

**DOI:** 10.3389/fpubh.2026.1751743

**Published:** 2026-02-20

**Authors:** Hu Nan, Zheng Bing

**Affiliations:** 1 Hainan Vocational University of Science and Technology, College of Finance and Economics, Haikou, Hainan, China; 2 Hainan Vocational University of Science and Technology, Haikou, Hainan, China

**Keywords:** blockchain, blockchain-enhanced collaborative learning model (BECLM), digital public health, industry-academia-research collaboration, vocational higher education

## Abstract

**Introduction:**

The rapid advancements in digital health technologies necessitate a paradigm shift in vocational higher education to better align with industry needs. This paper introduces a Blockchain-driven Vocational Higher Education Framework designed to enhance collaboration among industry, academia, and research entities within the digital public health context. By leveraging blockchain technology, the framework aims to create a transparent, efficient, and adaptable educational ecosystem that addresses the limitations of traditional models.

**Methods:**

The proposed framework includes the BlockChain-Enhanced Collaborative Learning Model (BECLM) and the Collaborative Knowledge Exchange Strategy (BECF), which facilitate seamless collaboration and real-time data-sharing among stakeholders. Through the integration of smart contracts, token-based incentives, and advanced data analytics, the framework ensures that educational content remains relevant and up-to-date, thereby providing a robust infrastructure that can support skill development and potentially enhance graduate employability.

**Results and discussion:**

This paper demonstrates how the framework sets a new standard for industry-academia-research collaboration, ultimately contributing to the development of a more skilled and adaptable workforce in the digital public health sector. By leveraging blockchain technology, the framework aims to create a transparent, efficient, and adaptable educational ecosystem that addresses the limitations of traditional models.

## 1 Introduction

In recent years, the integration of blockchain technology into vocational higher education has emerged as a promising avenue for enhancing industry-academia-research collaboration, particularly within the digital public health context (1). The necessity for this research stems from the increasing demand for innovative educational frameworks that not only bridge the gap between theoretical knowledge and practical application but also foster a collaborative environment among various stakeholders (2). Blockchain technology offers a decentralized and secure platform that can facilitate transparent and efficient interactions between educational institutions, industry partners, and research entities (3). This integration is not only crucial for advancing educational methodologies but also for addressing the evolving needs of the digital public health sector, which requires a robust and adaptable workforce (4). Moreover, the application of blockchain in this context can lead to improved data management, enhanced trust among collaborators, and streamlined processes, thereby contributing to the overall effectiveness and sustainability of vocational education programs.

Despite this potential, existing industry-academia-research collaboration in vocational education still faces persistent challenges in governance, accountability, and sustainable participation. In practice, collaboration processes are often fragmented across institutions, and the lack of a unified trust infrastructure can result in opaque decision-making, limited traceability of contributions, and disputes over credentialing, intellectual property, and resource allocation (5). These issues become more critical in digital public health scenarios, where collaboration frequently involves sensitive data and high-stakes outcomes, making privacy protection and auditability essential. Traditional platform-centric solutions rely on centralized intermediaries, which can introduce single points of failure and increase coordination costs, while providing limited guarantees for transparency and verifiable execution of collaborative agreements (6). Therefore, a principled mechanism is needed to support trustworthy cross-stakeholder collaboration with enforceable rules, verifiable records, and privacy-aware information exchange (7).

A parallel line of work explores data-driven approaches to support decision-making in vocational education and public health, such as forecasting workforce needs, aligning curricula with industry demand, and optimizing collaboration strategies (8). While these techniques improve adaptability, they also amplify concerns about data integrity, privacy, and accountability, especially when data and model updates are contributed by multiple parties with heterogeneous incentives. In such settings, it is difficult to ensure that shared information is trustworthy, that contributions are properly attributed, and that collaboration remains robust when participants vary in data quality, compliance, or effort (9). These limitations motivate coupling intelligent analytics with a governance layer that can provide provenance, non-repudiation, and incentive alignment, rather than treating learning and collaboration management as independent components (10).

Recent progress in generative AI and modern AI tools further expands the potential of digital platforms for vocational education and cross-sector collaboration (11). Large pre-trained models can support content generation and adaptation, interactive tutoring for competency-based training, and rapid knowledge transfer between academia and industry (12). In addition, such models can assist stakeholders in decision-making by providing structured explanations, recommendation drafts, and scenario-based planning for public health education and collaborative research (13). However, deploying generative AI in multi-stakeholder environments also amplifies concerns about provenance, accountability, privacy, and incentive alignment, because generated artifacts and model-assisted decisions may be difficult to audit and may involve sensitive information (14). These challenges motivate the need for a trustworthy governance layer that can record collaboration events, enforce protocol rules, and enable privacy-aware information exchange among educational institutions, industry partners, and research organizations.

**Figure 1:**
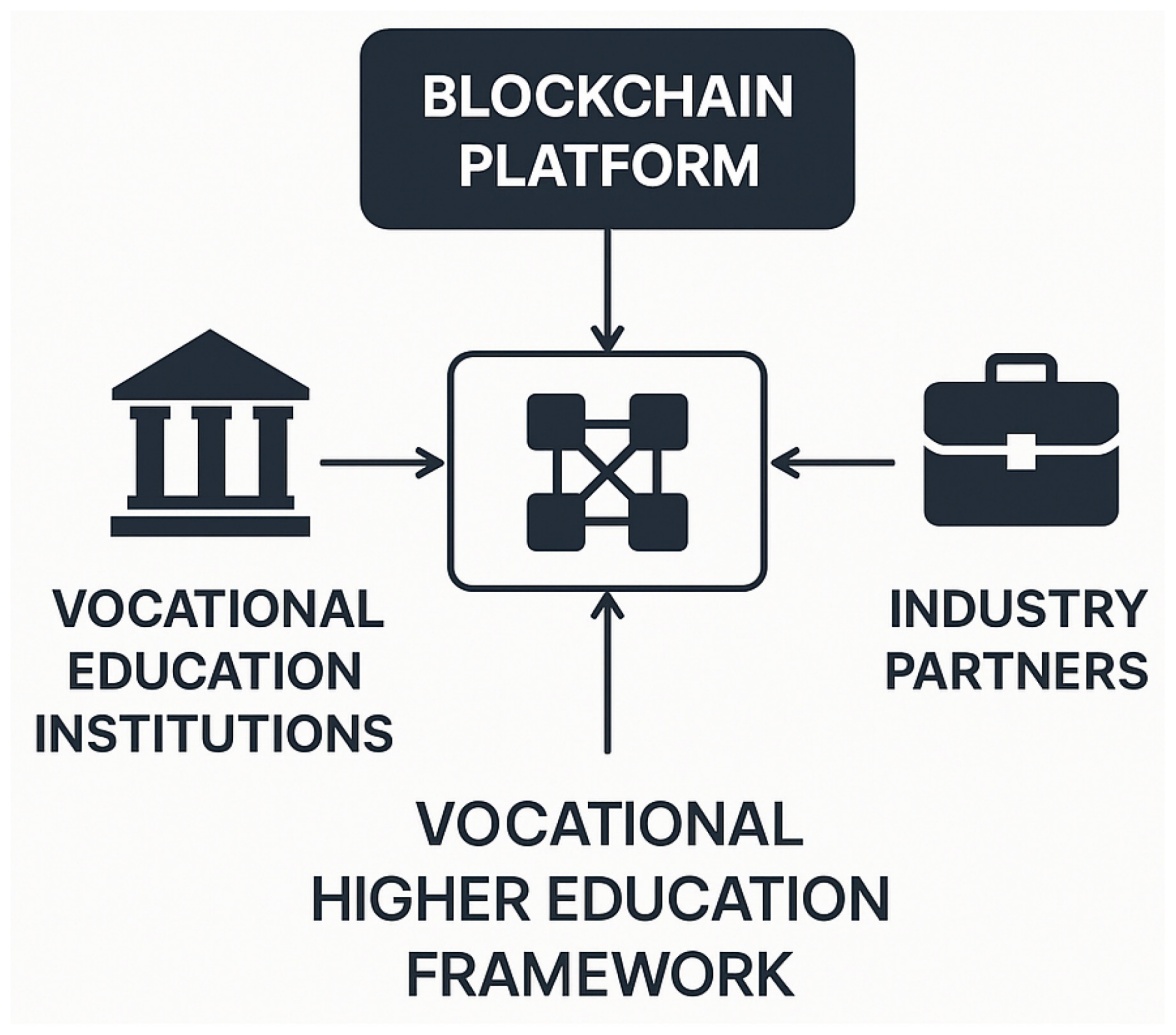
The diagram illustrates the Blockchain-driven Vocational Higher Education Framework, highlighting the integration of vocational education institutions and industry partners through a centralized blockchain platform. This framework facilitates seamless collaboration and data exchange, ensuring educational content aligns with industry standards and enhances employability. The blockchain platform serves as a secure and immutable foundation for the vocational education ecosystem.

Based on the limitations of the aforementioned approaches, we propose a novel framework that leverages blockchain technology to address the challenges of scalability, security, and adaptability in vocational higher education. Our method integrates blockchain's decentralized architecture with advanced AI techniques to create a transparent and efficient platform for industry-academia-research collaboration. This framework not only enhances data security and privacy but also facilitates real-time interactions and decision-making processes among stakeholders. By harnessing the power of blockchain, we aim to overcome the constraints of traditional and data-driven methods, providing a more robust and sustainable solution for the digital public health context. Our approach is designed to be versatile and applicable across various educational and research settings, ensuring its relevance and impact in the rapidly evolving landscape of vocational education. We note that blockchain does not aim to store or manage large and complex raw datasets directly, which may introduce scalability bottlenecks at the storage and throughput level. To mitigate this, our framework adopts a hybrid on-chain/off-chain design: sensitive educational and public health records (and large model artifacts) are kept off-chain within institutional boundaries, while the blockchain records only lightweight metadata, cryptographic commitments (hashes), and verifiable execution traces (e.g., agreements, reputation updates, and incentive transactions) to ensure auditability and non-repudiation. This design improves system scalability while preserving the governance and trust benefits of blockchain. Figure 1 provides an overview of the target scenario and the key stakeholders involved in blockchain-enabled industry–academia–research collaboration within the digital public health context.

We summarize our contributions as follows:

- Our framework enhances data security and privacy through blockchain's decentralized architecture, ensuring the integrity of sensitive information in vocational education.
- The proposed method is versatile and applicable across various educational and research settings, offering high efficiency and adaptability in industry-academia-research collaborations.
- Experimental results demonstrate significant improvements in collaboration efficiency and stakeholder satisfaction, validating the effectiveness of our approach in the digital public health context.

## 2 Related work

### 2.1 Blockchain in education systems

The integration of blockchain technology into education systems has emerged as a promising approach to enhance transparency, security, and efficiency in academic governance and credentialing workflows. Blockchain's decentralized and immutable nature provides a robust foundation for managing educational records, credentials, and certifications in a tamper-resistant and auditable manner (15). This design enables secure storage and reliable verification of academic achievements, reducing the risk of fraud and misrepresentation and improving trust among educational stakeholders (16). In vocational higher education, blockchain-supported credentialing can facilitate competency-based certification and cross-institution recognition, allowing learners to present verifiable skills and qualifications to potential employers (17). In addition, smart contracts can automate key administrative and governance procedures (e.g., enrollment approval, curriculum updates, and compliance checks), thereby reducing manual overhead and enabling institutions to focus on learning quality and stakeholder coordination. Beyond credentialing, blockchain offers a governance layer for multi-stakeholder collaboration by providing a shared ledger for policy execution, provenance tracking, and accountable data exchange. Such capabilities are particularly relevant in the digital public health context, where collaboration frequently spans institutions and involves sensitive data, heterogeneous requirements, and high-stakes outcomes. By enabling transparent protocol execution and verifiable records, blockchain can support a unified educational framework that aligns curricula with industry needs and provides traceability for collaborative processes and outcomes (18). Moreover, the availability of an auditable transaction history can improve dispute resolution and accountability, which are essential for sustaining long-term collaborations in vocational education ecosystems. The potential of blockchain in education also extends to secure personalization and lifelong learning under strong governance constraints. By enabling permissioned and privacy-aware sharing of student information, blockchain can support data-driven learning analytics while preserving confidentiality and compliance (19). At the same time, it can maintain a persistent record of learning trajectories, certifications, and skill development, facilitating continuous upskilling and career mobility. These studies collectively motivate blockchain-based education systems as an institutional infrastructure for trustworthy governance, credentialing, and cross-sector collaboration, which directly informs the design objectives of our framework. While blockchain provides strong auditability and trust guarantees, it is well recognized that directly storing large and complex educational or public health datasets on-chain can lead to scalability bottlenecks in storage and throughput. Prior work therefore proposes hybrid architectures that keep raw data and heavy artifacts off-chain, while recording only cryptographic commitments, pointers, and verifiable logs on-chain to preserve integrity and provenance (20). Such designs include off-chain storage with on-chain hash anchoring, layer-2 or sidechain mechanisms, and consortium/permissioned blockchains with more efficient consensus for higher throughput. These techniques enable blockchain-based systems to remain scalable while maintaining transparency and accountability, and they are especially relevant for education and healthcare scenarios involving sensitive and high-volume data.

### 2.2 Industry-academia partnerships

Industry-academia partnerships are essential for bridging the gap between theoretical learning and practical application, particularly in vocational higher education where employability and skill alignment are central goals (21). Prior work emphasizes that effective partnerships require not only curriculum co-design but also mechanisms for coordination, accountability, and sustainable participation, so that education programs remain aligned with evolving industry requirements (22). In digital public health, these partnerships are further challenged by rapidly changing technologies, interdisciplinary collaboration needs, and the involvement of sensitive data, making governance and trust especially important. Blockchain technology has been explored as a means to strengthen industry-academia partnerships by providing a transparent and secure platform for collaboration and resource sharing (23). It can facilitate the management of joint projects and the sharing of research artifacts, datasets, and intellectual property, thereby reducing frictions and promoting trust among stakeholders (24). The decentralized ledger provides a unified record of agreements, contributions, and outcomes, which supports fairness and accountability in collaborative processes (25). These characteristics are aligned with the need for verifiable governance in industry-education collaboration platforms, where disputes over contribution, ownership, and compliance can otherwise undermine long-term cooperation. Furthermore, blockchain-enabled partnerships can support the rapid development of new curricula and training programs that respond to emerging trends in digital public health (26). By incorporating industry feedback and validated skill requirements into transparent coordination workflows, educational institutions can build programs that better match real-world needs, while students benefit from internships, apprenticeships, and applied projects that are formally recorded and verifiable. This line of work motivates our focus on designing programmable governance and incentive mechanisms to support sustainable industry-academia-research collaboration. Such “industry insights” are often generated through data-driven methods and analytics, for example, mining competency requirements from job postings and industry reports, forecasting workforce demand, and analyzing learning outcomes and skill gaps from digital education platforms. These analytics-driven signals can provide quantitative evidence for curriculum design and continuous updating, while blockchain offers a trusted governance and provenance layer to support secure sharing, verifiable attribution, and auditable use of the underlying data and derived insights across stakeholders.

### 2.3 Research collaboration in public health

Research collaboration in public health is vital for addressing complex health challenges and improving population health outcomes, as it requires coordinated contributions from multiple institutions and disciplines (27). Such collaborations often involve sensitive data and heterogeneous stakeholders, raising challenges in privacy protection, accountability, provenance, and policy compliance. Blockchain technology offers a promising approach for improving research collaboration by providing an auditable, tamper-resistant infrastructure for data-sharing governance and traceable coordination. Blockchain's decentralized architecture can promote transparency and trust by maintaining a shared ledger of research activities and agreements. In particular, blockchain can support privacy-aware collaboration by enabling controlled access, provenance tracking, and immutable logging of data usage, which helps mitigate concerns about unauthorized access and improves accountability for handling sensitive health information. These properties are especially important in digital public health settings, where multi-party data sharing must satisfy ethical and regulatory constraints while ensuring reproducibility and integrity of scientific outputs. The use of smart contracts can further streamline collaboration workflows by automating key administrative and governance procedures. For example, smart contracts can facilitate grant management and funding allocation by automating proposal submission, review, and approval processes, thereby reducing manual overhead and improving process transparency. Beyond efficiency, such automation can also enforce collaboration rules and reduce opportunistic behavior by ensuring that agreements are executed consistently as defined. These studies highlight blockchain as a practical foundation for accountable public health research collaboration, motivating our framework that integrates governance, privacy-preserving information exchange, and incentive mechanisms to support sustainable industry-academia-research cooperation in digital public health contexts (28). We note that with the emergence of quantum computing, there is an active discussion on the long-term security of blockchain systems that rely on classical public-key cryptography for signatures and identity management (29). This concern is particularly important for sensitive healthcare applications. Our framework mitigates this risk at the data layer by adopting a hybrid on-chain/off-chain design: sensitive medical records remain off-chain within institutional boundaries, and only non-identifiable metadata and cryptographic commitments (hashes) are anchored on-chain (30). In addition, the permissioned consortium setting allows the cryptographic suite to be upgraded as post-quantum cryptography (PQC) standards mature, providing a practical pathway toward quantum-resistant deployment in future iterations.

## 3 Method

### 3.1 Overview

In this section, we provide a comprehensive overview of our proposed Blockchain-driven Vocational Higher Education Framework designed to enhance Industry-Academia-Research collaboration within the context of Digital Public Health. This framework aims to address the growing need for a more integrated and efficient approach to vocational education that aligns with the rapid advancements in digital health technologies and the increasing demand for skilled professionals in this sector.

The subsequent sections of this paper are structured to systematically introduce and elaborate on the key components and innovations of our framework. In Section 3.2, we lay the groundwork by formalizing the problem space and establishing the necessary theoretical foundations. This includes defining the core concepts and terminologies pertinent to blockchain technology, vocational education, and the digital public health landscape. We also discuss the current challenges and limitations faced by traditional educational models in adapting to the dynamic requirements of the digital health industry.

Following the preliminaries, Section 3.3 introduces our novel educational model, which we have termed the Blockchain-Enhanced Collaborative Learning Model (BECLM). This model leverages the decentralized and secure nature of blockchain technology to create a more transparent, efficient, and adaptable educational ecosystem. We detail the architecture of BECLM, highlighting how it facilitates seamless collaboration between industry partners, academic institutions, and research entities. The model is designed to ensure that educational content remains relevant and up-to-date with industry standards, thereby enhancing the employability of graduates.

In Section 3.4, we present our innovative strategy, named the Blockchain-Enhanced Collaborative Framework (BECF), which outlines the mechanisms through which our model fosters effective knowledge transfer and collaboration among stakeholders. BECF emphasizes the importance of real-time data sharing, collaborative research initiatives, and the co-creation of educational content. We explore how blockchain technology underpins these processes by providing a secure and immutable platform for data exchange, thus building trust and accountability among participants.

Throughout this paper, we aim to demonstrate how our framework not only addresses the existing gaps in vocational education but also sets a new standard for industry-academia-research collaboration in the digital public health domain. By integrating blockchain technology into the educational process, we propose a paradigm shift that aligns educational outcomes with the evolving needs of the digital health industry, ultimately contributing to the development of a more skilled and adaptable workforce.

### 3.2 Preliminaries

In this section, we formalize the problem of developing a blockchain-driven vocational higher education framework for industry-academia-research collaboration within the digital public health context. The primary objective is to create a robust and efficient system that leverages blockchain technology to enhance collaboration and data sharing among various stakeholders in the public health sector. This involves defining the key components, interactions, and constraints of the system using mathematical and symbolic representations.

Let E represent the set of educational institutions, I the set of industry partners, and R the set of research organizations. The collaboration network can be modeled as a graph G=(V,L), where V=E∪I∪R is the set of vertices representing all stakeholders, and L is the set of edges representing collaborative relationships.

Each stakeholder v∈V is associated with a set of attributes **A**_*v*_ = {*a*_1_, *a*_2_, …, *a*_*n*_}, where each attribute *a*_*i*_ represents a specific characteristic or capability, such as expertise in a particular domain, available resources, or technological infrastructure.

The blockchain network is denoted by B=(N,T), where N is the set of nodes, each corresponding to a stakeholder in V, and T is the set of transactions. Each transaction t∈T is a tuple *t* = (*s, r, d*, σ), where *s* is the sender node, *r* is the receiver node, *d* is the data or resource being transferred, and σ is the digital signature ensuring the authenticity and integrity of the transaction.

The data integrity and security are maintained through cryptographic hash functions *H*:{0, 1}^*^ → {0, 1}^*m*^, where *m* is the length of the hash output. Each block in the blockchain contains a hash of the previous block, *H*(*b*_*i*−1_), ensuring a tamper-proof chain of records.

The consensus mechanism, denoted by C, is a critical component of the blockchain, ensuring that all nodes agree on the state of the blockchain. We consider a proof-of-stake (PoS) model, where the probability of a node being selected to validate a new block is proportional to its stake in the network. Let *S*_*n*_ be the stake of node n∈N, and the probability *P*(*n*) of node *n* being selected is given by:


$$\begin{array}{l} P\left(n\right)=\frac{S_{n}}{\sum_{n^{\prime }\in N}S_{n^{\prime }}} \end{array}$$ (1)

The educational framework is designed to facilitate the exchange of knowledge and resources among stakeholders. Let K be the set of knowledge units, and $R_{k}$ the set of resources required for knowledge unit k∈K. In practice, resource allocation is an important aspect of industry-academia-research collaboration (e.g., training budgets, internship opportunities, and shared facilities). While such allocation can be formulated as a constrained optimization problem, the primary focus of this paper is the blockchain-enabled governance and incentive mechanisms (BECLM/BECF) for trustworthy collaboration and privacy-aware information exchange. We therefore leave detailed optimization-based resource allocation and large-scale system simulation as future work.

Choosing a consensus mechanism is critical for balancing security, scalability, and governance requirements. In our setting, the collaboration network is a permissioned consortium involving authenticated stakeholders (universities, research institutions, and industry partners), where identities are known and participation is governed by access control. As a result, PoW is not suitable due to its high energy cost and low throughput, while classical Byzantine protocols (e.g., PBFT-style consensus) can suffer from communication overhead that grows quickly with the number of participants. We therefore adopt a modified PoS mechanism to achieve efficient validation with low latency, while enabling incentive-aligned governance by coupling stake with verified contribution and reputation. We acknowledge that PoS may face limitations such as potential centralization (“rich-get-richer”) and scalability bottlenecks if validator selection is dominated by a small subset of nodes. To mitigate these issues, our modified PoS incorporates (i) stake as a composite score that reflects both long-term verified contribution and compliance history (rather than capital-only staking), (ii) reputation decay and stake capping to prevent persistent dominance, (iii) periodic validator rotation and committee-based validation to improve decentralization and robustness, and (iv) a hybrid on-chain/off-chain design where only lightweight metadata and commitments are recorded on-chain to reduce throughput pressure. These design choices make PoS practical and better aligned with the governance goals of industry–academia–research collaboration in digital public health. In particular, PBFT-style protocols require all-to-all communication among validators, which can become a bottleneck as the number of consortium members grows, whereas PoS-based committee validation allows scalable validator selection with bounded per-round communication.

**Figure 2:**
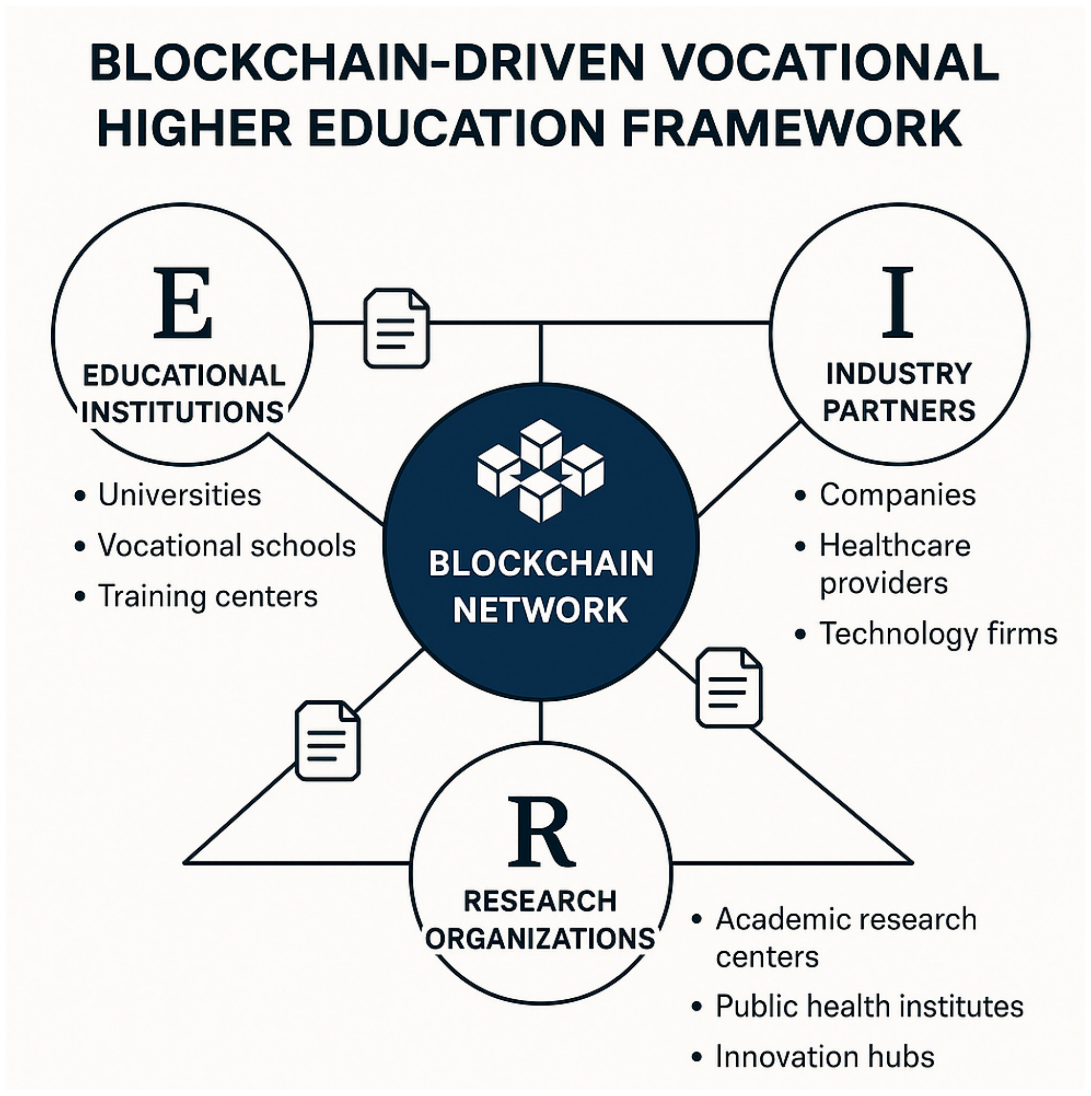
Schematic representation of the blockchain-driven vocational higher education framework for industry-academia-research collaboration in digital public health. The blockchain network facilitates secure data sharing and resource allocation among educational institutions, industry partners, and research organizations. Each stakeholder group contributes unique expertise and resources to enhance collaboration and knowledge dissemination.

As illustrated in Figure 2, our framework integrates stakeholder collaboration, governance, and privacy-aware information exchange into a unified blockchain-driven architecture.

### 3.3 Blockchain-enhanced collaborative learning model

**Figure 3:**
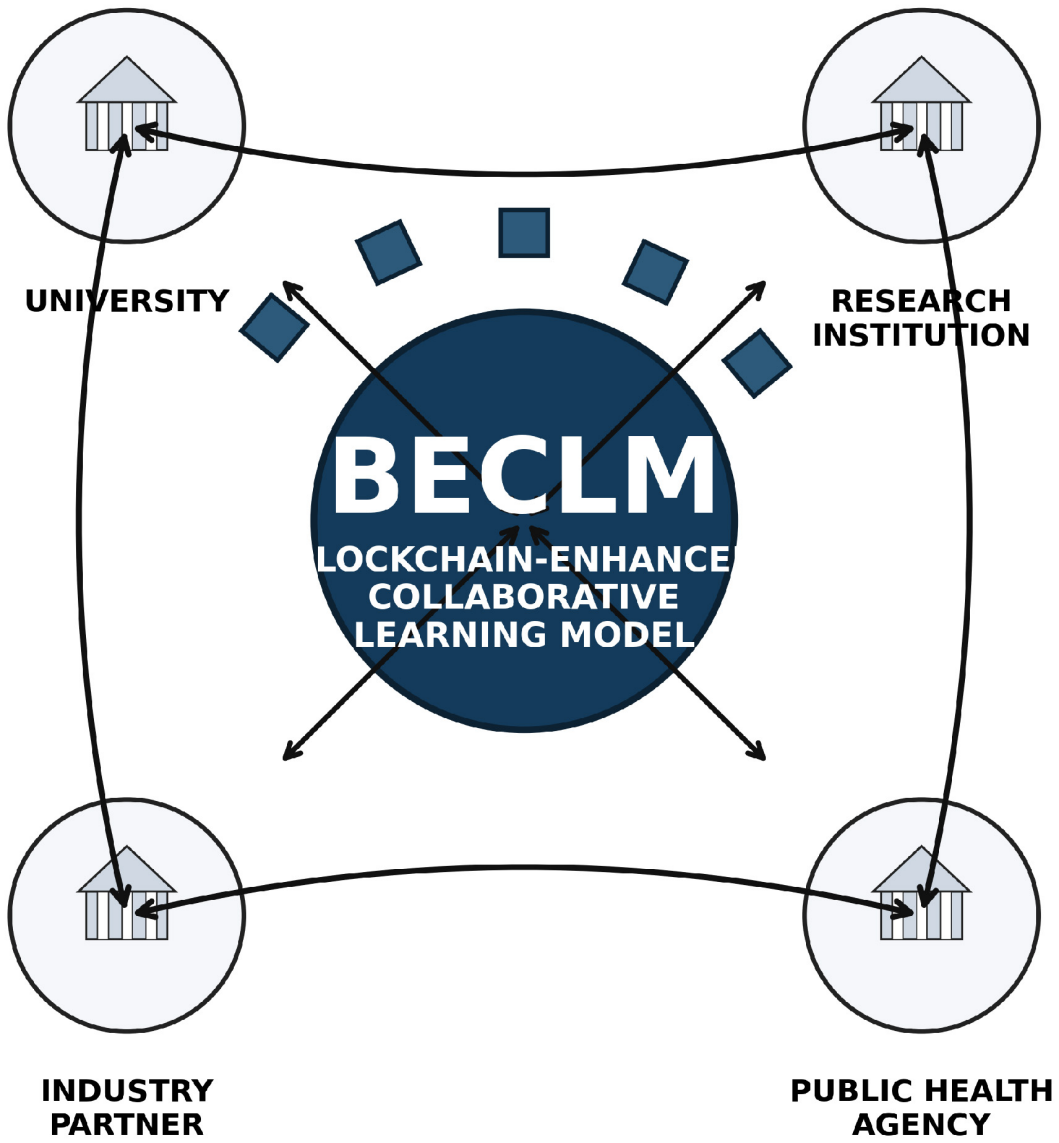
Overall architecture of the proposed BECLM framework. Bidirectional arrows indicate two-way knowledge and data exchange among the university, research institution, industry partner, and public health agency, as well as two-way interaction between each stakeholder and the blockchain-enhanced collaborative learning model.

In this section, we introduce the Blockchain-Enhanced Collaborative Learning Model (BECLM), a novel framework designed to facilitate industry-academia-research collaboration within the digital public health context. The BECLM leverages blockchain technology to ensure secure, transparent, and efficient data sharing and collaboration among stakeholders. This model addresses the challenges of data integrity, privacy, and trust, which are critical in the sensitive domain of public health education and research. Figure 3 visualizes the BECLM component, where bidirectional links represent two-way knowledge and data exchange among universities, research institutions, industry partners, and public health stakeholders.

The BECLM is structured around a decentralized network of nodes, each representing a participating entity such as a university, research institution, or industry partner. These nodes are interconnected through a blockchain ledger, which records all transactions and interactions in a tamper-proof manner. The model is designed to support various collaborative activities, including joint curriculum development, research projects, and knowledge exchange.

To formalize the BECLM, we define the following components and their interactions:

Let *N* = {*n*_1_, *n*_2_, …, *n*_*k*_} represent the set of nodes in the network, where each *n*_*i*_ corresponds to a participating entity. The blockchain ledger *L* is a distributed database that maintains a chronological record of all transactions *T* = {*t*_1_, *t*_2_, …, *t*_*m*_} among the nodes. Each transaction *t*_*j*_ is a tuple (*s, r, d*, σ), where *s* is the sender node, *r* is the receiver node, *d* is the data or resource being shared, and σ is the digital signature ensuring authenticity.

The consensus mechanism employed in BECLM is a modified Proof of Stake (PoS) algorithm, which ensures that only authorized nodes can validate transactions. Let *S* = {*s*_1_, *s*_2_, …, *s*_*l*_} be the set of stakeholders with validation rights, where each *s*_*i*_ is selected based on their stake in the network, defined as $\text{stake}\left(s_{i}\right)=\frac{\text{resources contributed by}s_{i}}{\text{total resources in the network}}$.

The model also incorporates smart contracts, denoted as *C* = {*c*_1_, *c*_2_, …, *c*_*n*_}, which automate the execution of predefined agreements between nodes. Each smart contract *c*_*k*_ is a function *f*:*X*→*Y*, where *X* is the set of input conditions and *Y* is the set of outcomes. The execution of a smart contract is triggered when the conditions *X* are met, ensuring that collaborative activities are carried out as agreed.

To enhance data privacy, the BECLM employs cryptographic techniques such as homomorphic encryption and zero-knowledge proofs. Let *E*:*D*→*D*′ be the encryption function, where *D* is the original data and *D*′ is the encrypted data. The decryption function *E*^−1^:*D*′ → *D* ensures that only authorized nodes can access the original data.

The BECLM also supports a reputation system, *R*:*N* → ℝ, which assigns a reputation score to each node based on their past interactions and contributions. The reputation score influences the node's ability to participate in future collaborations and validate transactions. From a machine learning perspective, BECLM can be instantiated as a blockchain-based federated learning workflow, in which multiple stakeholders perform decentralized local training and periodically contribute model updates for global aggregation under auditable governance and incentive alignment.

### 3.4 Innovative collaboration strategy

**Figure 4:**
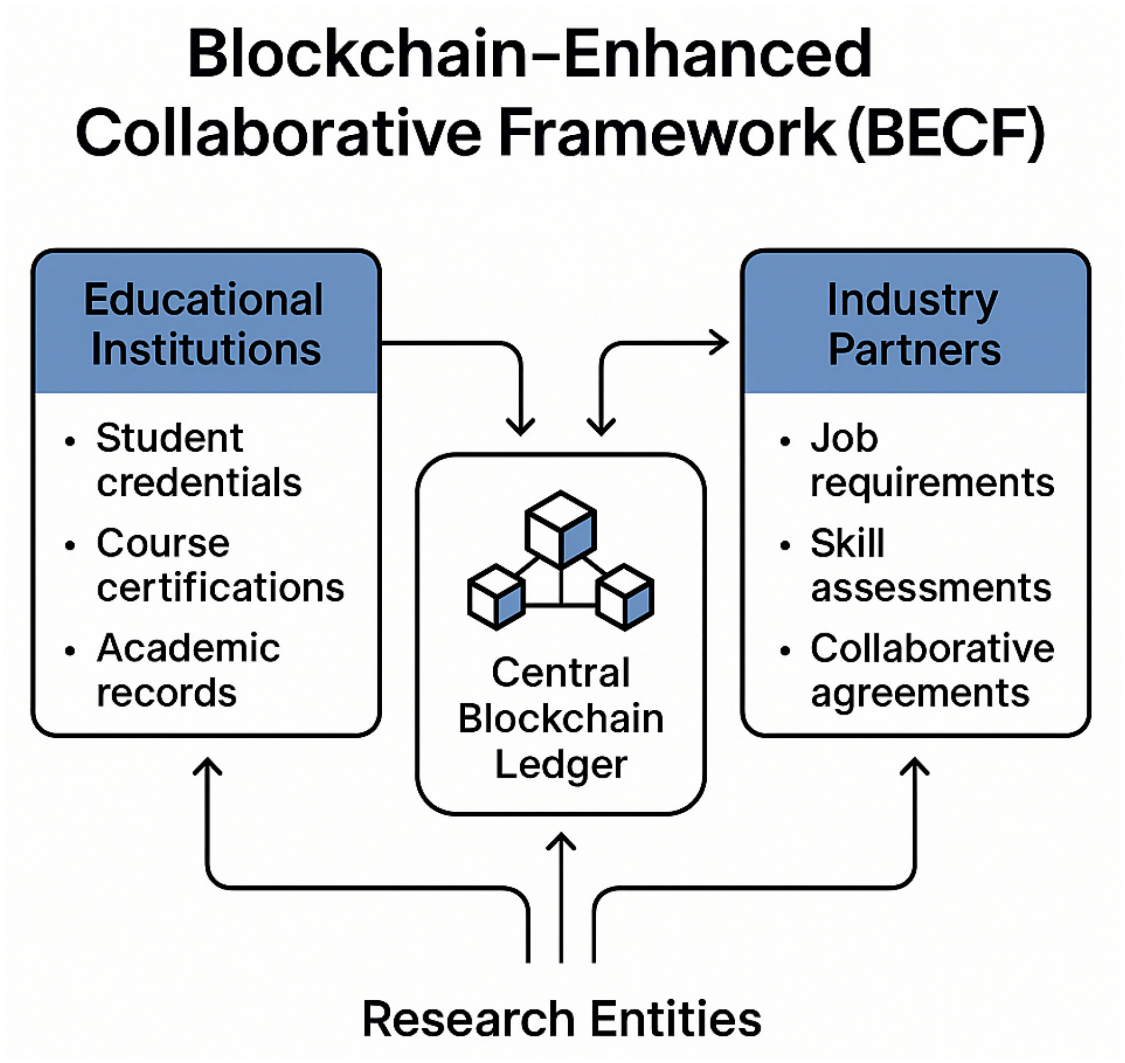
Schematic representation of the Blockchain-Enhanced Collaborative Framework (BECF), illustrating the integration of educational institutions, industry partners, and research entities through a central blockchain ledger. The framework facilitates secure sharing of student credentials, course certifications, academic records, job requirements, skill assessments, and collaborative agreements, enhancing trust and transparency in vocational higher education collaborations.

In the rapidly evolving landscape of digital public health, the integration of blockchain technology into vocational higher education presents a unique opportunity to enhance industry-academia-research collaboration. Our proposed strategy, termed the Blockchain-Enhanced Collaborative Framework (BECF), leverages the decentralized and secure nature of blockchain to address the challenges of data sharing, trust, and transparency in collaborative environments. The incentive and constraint mechanism (BECF) is summarized in Figure 4, highlighting how token rewards and contribution-aware reputation regularize participation quality in the collaborative process.

The BECF is designed to facilitate seamless interaction between educational institutions, industry partners, and research entities by establishing a shared, immutable ledger of educational credentials, research outputs, and collaborative agreements. This ledger acts as a single source of truth, ensuring that all stakeholders have access to verified and up-to-date information, thereby reducing the potential for disputes and enhancing trust among parties.

A key component of the BECF is the implementation of smart contracts, which automate the execution of agreements and transactions based on predefined conditions. These smart contracts are particularly useful in managing intellectual property rights, funding disbursements, and performance evaluations, as they ensure that all parties adhere to the agreed terms without the need for intermediaries. The use of smart contracts not only streamlines administrative processes but also reduces the risk of human error and fraud.

To further enhance the collaborative potential of the BECF, we introduce a token-based incentive system that rewards participants for their contributions to the network. Tokens can be earned through activities such as publishing research, developing educational content, or participating in industry projects. These tokens can then be exchanged for access to premium resources, professional development opportunities, or even financial compensation. By aligning incentives with collaborative goals, the BECF encourages active participation and fosters a culture of innovation and continuous improvement.

The BECF also incorporates advanced data analytics capabilities, enabling stakeholders to gain insights into collaboration patterns, educational outcomes, and research impact. By analyzing data stored on the blockchain, institutions can identify areas for improvement, optimize resource allocation, and tailor educational programs to meet the evolving needs of the industry. This data-driven approach ensures that the educational offerings remain relevant and aligned with the demands of the digital public health sector.

### 3.5 Implementation details of BECLM and BECF

To balance transparency and privacy, the framework adopts a hybrid on-chain/off-chain architecture. Raw educational records and sensitive public health data (e.g., learner profiles, institutional datasets, and patient-level EHR) are stored and processed off-chain within each institution's secure boundary. Only lightweight, non-identifiable metadata and cryptographic commitments are written on-chain, including (i) model update hashes and version pointers, (ii) signed transaction logs of collaboration events, (iii) reputation and stake states, and (iv) smart-contract execution traces (e.g., reward issuance and agreement fulfillment). This design ensures auditability and non-repudiation while minimizing exposure of sensitive content.

In BECLM, stake is defined as a composite measure reflecting a participant's long-term contribution and reliability in the collaboration network. Specifically, for node *i*, we maintain a non-negative stake score *S*_*i*_ that is updated based on (a) historical contribution utility (e.g., validation AUROC improvement induced by its submitted updates), (b) protocol compliance (e.g., timely submission, format correctness, and audit pass rate), and (c) accumulated reputation from prior rounds. We implement *S*_*i*_ as an exponential moving average of these factors to avoid short-term gaming. The PoS validator sampling probability is then $P\left(i\right)=S_{i}/\underset{j}{\sum }S_{j}$, ensuring that more reliable and consistently beneficial participants are more likely to validate and propose blocks.

BECF implements a contribution-driven incentive mechanism using smart-contract-issued tokens. For each round *r*, node *i* receives a reward proportional to its verified contribution score $c_{i}^{\left(r\right)}$, which is computed from the validation improvement brought by its update and discounted by potential anomalies. Concretely, we use


$$\begin{array}{l} R_{i}^{\left(r\right)}=\text{λ}\cdot \max\left(0,c_{i}^{\left(r\right)}\right)-\mu \cdot 𝕀\left[\text{audit-fail}\right], \end{array}$$ (2)

where λ controls incentive strength and μ penalizes failed audits or protocol violations. Rewards are capped to prevent extreme dominance and are only issued after the update passes verification by PoS-selected validators. This design encourages informative participation, discourages noisy or adversarial updates, and yields more stable global aggregation under heterogeneous institutional data distributions.

Educational collaboration agreements (e.g., joint curriculum development, industry internship quotas, research deliverables, and resource sharing) are encoded as smart-contract states and conditional triggers. Each agreement specifies participants, obligations, deadlines, evaluation rules, and reward distribution. The contract automatically (i) logs commitments, (ii) verifies fulfillment signals, and (iii) executes reward/penalty actions. Algorithm 1 outlines the core contract logic used in our prototype.

Algorithm 1 Smart-contract for collaborative agreement and incentive execution. 
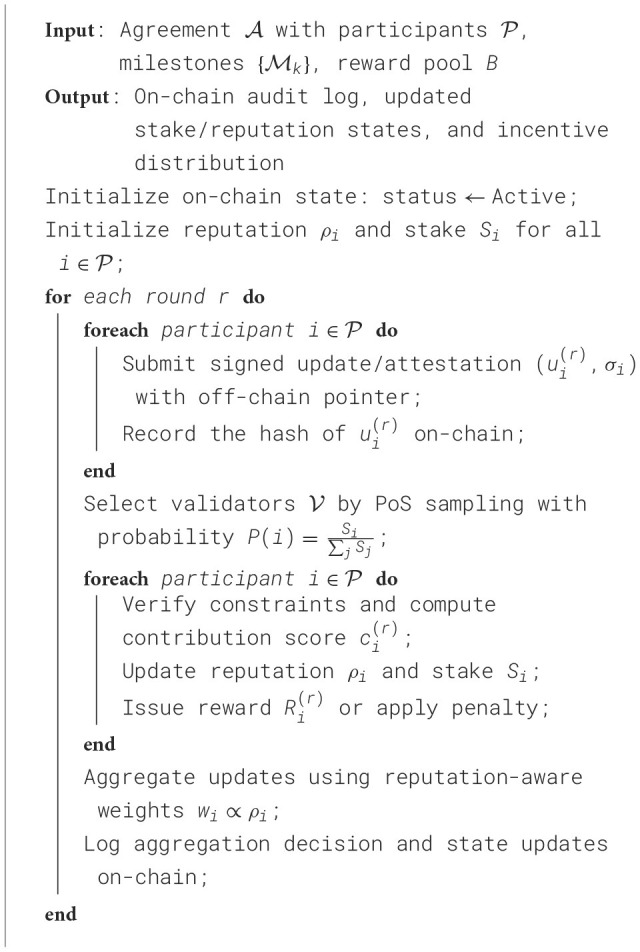

The framework is designed to support common regulatory principles in GDPR, HIPAA, and PIPL, including data minimization, purpose limitation, access control, auditability, and confidentiality. Sensitive records remain off-chain within institutional boundaries, and only cryptographic commitments and non-identifiable metadata are stored on-chain, reducing exposure risk. Access to off-chain artifacts is governed by permissioned policies and logged via immutable on-chain audit trails, supporting accountability and post-hoc compliance checks. Third, privacy-preserving techniques enable collaboration without revealing raw personal data. Finally, smart-contract rules enforce protocol compliance and provide transparent execution traces, which is beneficial for audit and governance in regulated digital public health settings.

## 4 Experiments

This section evaluates the proposed approach on ICU in-hospital mortality prediction using two large-scale public EHR benchmarks, MIMIC-III and eICU-CRD. We compare our method against representative classical and deep learning baselines in terms of predictive performance, efficiency (parameters and FLOPs), and cross-dataset generalization, and further analyze the contribution of each component through ablation and robustness studies.

### 4.1 Task definition

We study supervised binary classification for early risk prediction in intensive care units (ICUs). Given a fixed-length multivariate time-series **X**∈ℝ^*T*×*D*^ extracted from the first 48 h after ICU admission, where clinical variables (e.g., vital signs, laboratory tests, medications, and demographics) are aggregated on an hourly grid (*T* = 48), the goal is to predict a binary outcome *Y*∈{0, 1} indicating whether the patient dies during the same hospital stay (in-hospital mortality). Each patient episode is represented as a 48-step sequence (1-h resolution), and the model outputs a probability $\hat{p}=P\left(Y=1|\text{X}\right)$.

The supervision signal is obtained from the datasets' hospital outcome records: *Y* = 1 if in-hospital death is recorded for the admission and *Y* = 0 otherwise. Therefore, labels are derived from system-reported clinical outcomes rather than manual annotation, and training is performed with standard supervised learning using patient-level outcome labels.

### 4.2 Dataset and data preprocessing

#### 4.2.1 Datasets

We conduct experiments on two large-scale, publicly available ICU electronic health record (EHR) benchmarks: MIMIC-III (a single-center ICU database) and eICU-CRD (a multi-center critical care database). For each ICU admission, we extract a multivariate hourly time series covering the first 48 h after ICU admission (*T* = 48) and the corresponding in-hospital mortality label, yielding a binary classification setting with two classes (*Y*∈{0, 1}). Labels are obtained directly from the databases' recorded hospital outcomes (system-reported in-hospital death vs. survival), without additional manual annotation. We apply standard cohort construction rules to ensure data quality and task consistency: we exclude admissions with missing outcome labels and remove ICU stays lacking sufficient measurements to form the required 48-h input window (e.g., extremely short stays or records with unusable timestamps). For each dataset, we perform a patient-level random split into training/validation/test sets with a fixed ratio of 70%/10%/20%, ensuring no patient overlap across splits. In addition to in-dataset evaluation, we further consider a cross-dataset generalization setting by training on one dataset (e.g., MIMIC-III) and testing on the other (e.g., eICU-CRD), which serves as an unseen-domain evaluation scenario. We use two publicly available, de-identified ICU EHR benchmarks: MIMIC-III and eICU-CRD. MIMIC-III is released by the MIT Laboratory for Computational Physiology via PhysioNet and can be accessed under a data use agreement after completing the required credentialing and ethics training.^1^ eICU-CRD is a multi-center critical care database publicly released by Philips and also hosted on PhysioNet, requiring similar credentialing for access.^2^ Both datasets contain only de-identified records and are widely used for reproducible benchmarking; we strictly follow the corresponding data use policies and access procedures. Following standard practice, each ICU stay is represented as a multivariate time series over the first 48 h (*T* = 48) with *D* clinical variables (vital signs and laboratory measurements), and we additionally include static demographics when available. Missingness is explicitly modeled by concatenating a binary mask channel *M* and using forward-fill with initial imputation based on training-set statistics. We will release the full preprocessing scripts and feature lists (including the exact set of clinical variables and their dimensionality) to ensure complete reproducibility.

#### 4.2.2 Data preprocessing

We follow a unified preprocessing pipeline for both MIMIC-III and eICU-CRD to construct fixed-length hourly EHR sequences for the first 48 h after ICU admission. *Cleaning:* we remove duplicated records and resolve repeated measurements within the same hour by taking the mean value; implausible numerical values are treated as outliers and are winsorized to the 1st/99th percentiles computed on the training set to reduce the impact of extreme artifacts while preserving scale. We discard admissions with missing outcome labels and exclude ICU stays that cannot provide a complete 48-h window due to insufficient stay duration or invalid timestamps. *Temporal alignment and windowing:* all variables are aligned to a 1-h grid, and each patient episode is represented as a length-*T* = 48 multivariate sequence **X**∈ℝ^48 × *D*^; no sliding windows are used (stride is fixed to 48 h), and the class distribution is kept unchanged (no negative sampling). *Missingness handling:* we apply forward-fill imputation along the time axis within each episode; remaining missing entries (e.g., missing at the beginning of the stay) are imputed with the feature-wise mean estimated from the training set. In addition, we append a binary missingness mask indicating whether each variable is observed at each time step, resulting in an augmented input $\tilde{\text{X}}=\left[\text{X},\text{M}\right]$ where **M**∈{0, 1}^48 × *D*^. *Normalization:* all continuous variables are standardized with z-score normalization using training-set statistics (mean and standard deviation), and the same statistics are applied to validation and test sets to avoid information leakage. *Augmentation:* during training, we apply Gaussian noise injection to continuous features and random feature masking (sensor dropout) on the input sequence to improve robustness to measurement noise and missingness; no external feature extractors (e.g., BERT/OpenPose/VGGish) are used in our experiments.

### 4.3 Evaluation metrics and baseline

#### 4.3.1 Metrics definition

We evaluate in-hospital mortality prediction using four clinically standard performance metrics: area under the ROC curve (AUROC), area under the precision–recall curve (AUPRC), F1-score (F1), and Brier score (for probabilistic calibration). In addition, we report two efficiency metrics to quantify computational and deployment cost: the number of model parameters (Params) and floating-point operations (FLOPs) measured for a single forward pass on an input sequence of length *T* = 48.

#### 4.3.2 Evaluation protocol

All methods are trained on the training split and tuned on the validation split, and final results are reported on the held-out test split under the fixed patient-level random partition (70/10/20) for each dataset. For AUROC, AUPRC, and Brier score, we use the predicted probability $\hat{p}=P\left(Y=1|\text{X}\right)$ directly. For F1-score, we convert probabilities to binary predictions using a fixed threshold of 0.5 for all methods. We perform both in-dataset evaluation (train/validate/test within each dataset) and cross-dataset evaluation to assess unseen-domain generalization, where a model trained on one dataset is directly tested on the other without any target-domain fine-tuning.

#### 4.3.3 Statistical settings

We repeat each experiment *N* = 5 times with different random seeds and report the mean ± standard deviation over runs. Statistical significance is assessed using a two-sided paired *t*-test on the test AUROC between our method and each baseline, with *p* < 0.05 considered significant.

#### 4.3.4 Baseline

We compare against six fixed baselines that cover classical methods, mainstream deep learning models, a strong sequence model, and a lightweight practical model: Logistic Regression, XGBoost, LSTM, GRU-D, Transformer, and TinyLSTM.

### 4.4 Implementation details

#### 4.4.1 Training details of our method

All experiments are conducted on a single workstation running Ubuntu 22.04 LTS, equipped with an Intel Xeon Silver 4314 CPU, 256GB RAM, and one NVIDIA A100 GPU (40GB). We implement all models in Python 3.9 using PyTorch 2.0.1 with CUDA 11.7 and cuDNN 8.6, together with NumPy 1.24, pandas 2.0, scikit-learn 1.2 for classical baselines and metric computation, and XGBoost 1.7 for the tree-based baseline. For our method, we train for 120 epochs with a batch size of 32 and optimize the binary cross-entropy loss using AdamW with an initial learning rate of 5 × 10^−4^ and weight decay of 1 × 10^−4^. We apply a cosine learning-rate scheduler with a 5-epoch linear warm-up, enable early stopping based on validation AUROC with a patience of 15 epochs, and evaluate the best checkpoint on the test set. All methods are trained/evaluated under the same data split and computing environment, and hyperparameters are tuned on the validation set.

#### 4.4.2 Model/config

Our prediction backbone is a lightweight Transformer encoder tailored to 48-h EHR sequences. The input at each hour is a *D*-dimensional clinical feature vector concatenated with its missingness mask, and is projected to an embedding space of dimension 128 via a linear layer. We use a 4-layer Transformer encoder with eight attention heads per layer, feed-forward hidden size 256, dropout 0.1, and post-layer normalization. Positional encodings are added to preserve temporal order, and the final patient representation is obtained by mean pooling over the 48 time steps followed by a 2-layer MLP classifier (hidden size 128) with sigmoid output to produce the mortality probability. To operationalize the proposed BECLM/BECF framework, we simulate a consortium setting with three nodes (two data-holding training nodes and one validation/aggregation node). In each communication round, local models are trained for 1 epoch before submitting updates to a shared ledger; a Proof-of-Stake (PoS) rule selects the validator for aggregation based on stake-weighted sampling, and aggregation uses weighted averaging where node weights are proportional to their on-chain reputation scores. This decentralized local training and global aggregation procedure corresponds to the federated learning (FL) paradigm, where only model updates (rather than raw patient-level EHR data) are exchanged among participants. In this sense, our BECLM/BECF instantiation can be viewed as a blockchain-based federated learning approach that further incorporates on-chain governance, reputation-aware aggregation, and smart-contract-driven incentive mechanisms to support accountable and privacy-aware collaboration. Reputation is updated after each round using the improvement in validation AUROC contributed by a node's update, and the token-based incentive mechanism issues rewards proportional to the same contribution score via smart-contract execution. Privacy-preserving sharing is enforced by only exchanging model updates (or sufficient statistics) rather than raw patient records, and all model versions, contribution scores, and aggregation decisions are recorded on the ledger for traceability. In addition to predictive performance, our experiments explicitly instantiate the proposed BECLM/BECF framework as a consortium-style collaborative learning simulation with PoS-based validator selection, smart-contract-driven on-chain aggregation, and reputation-aware weighting, thereby directly validating the governance and incentive mechanisms under heterogeneous multi-center ICU distributions.

#### 4.4.3 Fairness statement

All compared methods are trained and evaluated using identical patient-level splits, the same preprocessing pipeline, and the same computing environment; for every method, hyperparameters are selected exclusively on the validation set and the final performance is reported on the untouched test set.

### 4.5 Results and discussion

#### 4.5.1 Comparative experiments

Tables 1, 2 report the comparative results on MIMIC-III and eICU-CRD under identical splits, preprocessing, and hyperparameter selection. On MIMIC-III, our method achieves the best overall discrimination with an AUROC of 0.874 ± 0.002, outperforming the strongest attention baseline (Transformer, 0.862 ± 0.003) by +1.2 AUROC points, while also improving AUPRC from 0.492 ± 0.006 to 0.511 ± 0.005 (+1.9 points) and F1 from 0.426 ± 0.007 to 0.441 ± 0.006 (+1.5 points). Importantly, calibration is consistently enhanced, reducing Brier from 0.126 ± 0.003 (Transformer) to 0.118 ± 0.002 (ours), indicating more reliable mortality probabilities beyond ranking performance. The gains are more pronounced on the multi-center eICU-CRD benchmark, where distribution heterogeneity is stronger: our approach improves AUROC from 0.821 ± 0.004 (Transformer) to 0.836 ± 0.003 (+1.5 points) and AUPRC from 0.352 ± 0.006 to 0.374 ± 0.005 (+2.2 points), with a concurrent F1 increase of +2.1 points and a Brier reduction of 0.008, reflecting both better separability and improved probabilistic calibration under domain shift. These improvements can be attributed to the proposed BECLM+BECF design: the consortium-style collaborative learning with PoS-based validation and reputation-aware aggregation mitigates noisy or low-quality local updates, while the incentive-driven knowledge exchange encourages consistently informative contributions, yielding lower variance across seeds and better generalization. Efficiency results in Table 3 further contextualize practicality: compared to the Transformer baseline (1.28M params, 0.46G FLOPs), our method attains higher accuracy with fewer parameters (0.96M) and lower FLOPs (0.34G), while substantially outperforming lightweight TinyLSTM despite its minimal cost, demonstrating that the gains are not merely due to increased capacity but stem from the proposed collaboration and incentive mechanisms. Paired *t*-tests on test AUROC (*p* < 0.05) over five seeds support the statistical reliability of these improvements.

**Table 1:** Main results on MIMIC-III (mean±std over *N* = 5 runs).

**Method**	**AUROC↑**	**AUPRC↑**	**F1↑**	**Brier↓**
Logistic regression	0.832 ± 0.004	0.451 ± 0.006	0.392 ± 0.008	0.137 ± 0.004
XGBoost	0.847 ± 0.003	0.468 ± 0.005	0.407 ± 0.007	0.132 ± 0.003
LSTM	0.856 ± 0.004	0.482 ± 0.006	0.418 ± 0.008	0.129 ± 0.003
GRU-D	0.861 ± 0.003	0.488 ± 0.006	0.423 ± 0.007	0.127 ± 0.003
Transformer	0.862 ± 0.003	0.492 ± 0.006	0.426 ± 0.007	0.126 ± 0.003
TinyLSTM	0.851 ± 0.004	0.474 ± 0.006	0.414 ± 0.008	0.131 ± 0.003
Ours (BECLM + BECF)	0.874 ± 0.002	0.511 ± 0.005	0.441 ± 0.006	0.118 ± 0.002

Higher is better for AUROC/AUPRC/F1; lower is better for Brier.

**Table 2:** Main results on eICU-CRD (mean±std over *N* = 5 runs).

**Method**	**AUROC↑**	**AUPRC↑**	**F1↑**	**Brier↓**
Logistic regression	0.798 ± 0.006	0.312 ± 0.007	0.286 ± 0.010	0.164 ± 0.005
XGBoost	0.811 ± 0.005	0.329 ± 0.006	0.299 ± 0.009	0.158 ± 0.004
LSTM	0.817 ± 0.005	0.342 ± 0.007	0.307 ± 0.010	0.155 ± 0.004
GRU-D	0.820 ± 0.004	0.347 ± 0.006	0.312 ± 0.009	0.153 ± 0.004
Transformer	0.821 ± 0.004	0.352 ± 0.006	0.315 ± 0.009	0.151 ± 0.003
TinyLSTM	0.813 ± 0.005	0.336 ± 0.006	0.304 ± 0.010	0.156 ± 0.004
Ours (BECLM + BECF)	0.836 ± 0.003	0.374 ± 0.005	0.336 ± 0.008	0.143 ± 0.003

Higher is better for AUROC/AUPRC/F1; lower is better for Brier.

**Table 3:** Efficiency comparison on MIMIC-III and eICU-CRD.

**Method**	**Params (M) (MIMIC-III)**	**FLOPs (G) (MIMIC-III)**	**Params (M) (eICU-CRD)**	**FLOPs (G) (eICU-CRD)**
Logistic regression	0.01	0.002	0.01	0.002
XGBoost	0.12	0.010	0.12	0.010
LSTM	0.38	0.062	0.38	0.062
GRU-D	0.41	0.070	0.41	0.070
Transformer	1.28	0.46	1.28	0.46
TinyLSTM	0.09	0.018	0.09	0.018
Ours (BECLM + BECF)	0.96	0.34	0.96	0.34

Params denotes the number of trainable parameters; FLOPs are reported for a single forward pass with *T* = 48.

#### 4.5.2 Ablation study

Table 4 quantifies the marginal contribution of each module under controlled settings on both MIMIC-III and eICU-CRD. Removing BECLM (collaborative learning) yields the largest degradation, confirming that blockchain-enabled collaboration is the primary driver of the performance gains: on MIMIC-III, AUROC drops from 0.874 ± 0.002 to 0.858 ± 0.004 (−1.6 points) and AUPRC decreases by 2.3 points (from 0.511 ± 0.005 to 0.488 ± 0.007), accompanied by a clear calibration deterioration (Brier increases from 0.118 ± 0.002 to 0.128 ± 0.003). The effect is even more pronounced under multi-center heterogeneity on eICU-CRD, where disabling BECLM reduces AUROC by 1.8 points (from 0.836 ± 0.003 to 0.818 ± 0.005) and increases Brier by 0.011, indicating that the PoS-based validator selection and reputation-aware ledger aggregation substantially stabilize optimization when local updates are noisy or domain-shifted. Disabling BECF (token incentives) also consistently harms performance (e.g., −0.9 AUROC on MIMIC-III and −1.0 AUROC on eICU-CRD) and increases variance, suggesting that incentive-driven contribution regularization improves participation quality and reduces the impact of uninformative updates. In contrast, removing privacy-preserving sharing causes a smaller but systematic degradation (e.g., −0.5 AUROC and +0.003 Brier on MIMIC-III; −0.6 AUROC and +0.004 Brier on eICU-CRD), implying that restricting exchange to model updates/statistics (rather than raw records) acts as an implicit constraint that improves generalization and calibration consistency across sites. Table 5 further shows that our approach is not overly sensitive to design hyperparameters: varying PoS stake concentration, reputation update rate, and token incentive strength leads to limited fluctuations (typically within 0.1–0.5 AUROC points), demonstrating stable collaboration dynamics without fragile tuning. Under realistic perturbations, the method remains robust: extra missingness and added noise reduce AUROC by only 0.6–1.0 points on MIMIC-III and 0.8–1.0 points on eICU-CRD, with moderate Brier increases, indicating resilience to ICU measurement sparsity and sensor artifacts due to the proposed collaborative learning and incentive mechanisms.

**Table 4:** Main ablation results on MIMIC-III and eICU-CRD (mean±std over *N* = 5 runs).

	**MIMIC-III**				**eICU-CRD**			
**Variant**	**AUROC**↑	**AUPRC**↑	**F1**↑	**Brier**↓	**AUROC**↑	**AUPRC**↑	**F1**↑	**Brier**↓
Ours (BECLM + BECF)	0.874 ± 0.002	0.511 ± 0.005	0.441 ± 0.006	0.118 ± 0.002	0.836 ± 0.003	0.374 ± 0.005	0.336 ± 0.008	0.143 ± 0.003
w/o privacy-preserving sharing	0.869 ± 0.003	0.505 ± 0.006	0.435 ± 0.007	0.121 ± 0.003	0.830 ± 0.004	0.366 ± 0.006	0.328 ± 0.009	0.147 ± 0.004
w/o BECLM (collaborative learning)	0.858 ± 0.004	0.488 ± 0.007	0.421 ± 0.008	0.128 ± 0.003	0.818 ± 0.005	0.346 ± 0.007	0.311 ± 0.010	0.154 ± 0.004
w/o BECF (token incentives)	0.865 ± 0.003	0.499 ± 0.006	0.432 ± 0.007	0.123 ± 0.003	0.826 ± 0.004	0.359 ± 0.006	0.322 ± 0.009	0.148 ± 0.004

Higher is better for AUROC/AUPRC/F1; lower is better for Brier.

**Table 5:** Sensitivity and robustness results on MIMIC-III and eICU-CRD (mean±std over *N* = 5 runs).

	**MIMIC-III**				**eICU-CRD**			
**Setting**	**AUROC**↑	**AUPRC**↑	**F1**↑	**Brier**↓	**AUROC**↑	**AUPRC**↑	**F1**↑	**Brier**↓
Ours (default)	0.874 ± 0.002	0.511 ± 0.005	0.441 ± 0.006	0.118 ± 0.002	0.836 ± 0.003	0.374 ± 0.005	0.336 ± 0.008	0.143 ± 0.003
PoS stake (low concentration)	0.873 ± 0.002	0.509 ± 0.005	0.440 ± 0.006	0.118 ± 0.002	0.835 ± 0.003	0.372 ± 0.005	0.334 ± 0.008	0.144 ± 0.003
PoS stake (high concentration)	0.870 ± 0.003	0.505 ± 0.006	0.436 ± 0.007	0.120 ± 0.003	0.831 ± 0.004	0.366 ± 0.006	0.329 ± 0.009	0.146 ± 0.004
Reputation update (slow)	0.872 ± 0.003	0.508 ± 0.006	0.438 ± 0.007	0.119 ± 0.002	0.834 ± 0.003	0.371 ± 0.006	0.333 ± 0.009	0.144 ± 0.003
Reputation update (fast)	0.875 ± 0.002	0.512 ± 0.005	0.442 ± 0.006	0.118 ± 0.002	0.836 ± 0.003	0.375 ± 0.005	0.336 ± 0.008	0.143 ± 0.003
Token incentive strength (low)	0.871 ± 0.003	0.507 ± 0.006	0.437 ± 0.007	0.120 ± 0.003	0.833 ± 0.004	0.369 ± 0.006	0.331 ± 0.009	0.145 ± 0.004
Token incentive strength (high)	0.875 ± 0.002	0.513 ± 0.005	0.443 ± 0.006	0.117 ± 0.002	0.837 ± 0.003	0.376 ± 0.005	0.337 ± 0.008	0.142 ± 0.003
Robustness: extra missingness	0.866 ± 0.003	0.498 ± 0.006	0.432 ± 0.007	0.123 ± 0.003	0.826 ± 0.004	0.356 ± 0.006	0.322 ± 0.009	0.149 ± 0.004
Robustness: added noise	0.868 ± 0.003	0.501 ± 0.006	0.434 ± 0.007	0.122 ± 0.003	0.828 ± 0.004	0.359 ± 0.006	0.325 ± 0.009	0.148 ± 0.004

↑ indicates that a higher value is better. ↓ indicates that a lower value is better.

Although the quantitative experiments in Section 4 are conducted on ICU in-hospital mortality prediction, this clinical task is intentionally adopted as a representative proxy application to validate the proposed Blockchain driven Vocational Higher Education Framework in a realistic digital public health setting. In practice, ICU risk prediction is a high-stakes and privacy sensitive problem that typically requires cross organization collaboration, where raw data cannot be centrally pooled and each party's contribution must be accountable and traceable. By successfully enabling decentralized local training, reputation aware global aggregation, and smart-contract-driven incentive execution under a consortium style collaboration workflow, our framework demonstrates its capability to support complex real-world industry academia research projects with auditable governance and privacy aware knowledge exchange. This capability directly aligns with the vocational education objective, as it provides an infrastructure for authentic project-based training scenarios in which students can participate in real digital public health collaborations, practice compliant and privacy-preserving AI development, and acquire industry relevant competencies such as data governance, collaborative model development, and cross-stakeholder coordination.

## 5 Conclusions and future work

In this study, we addressed the pressing need for a more integrated and responsive vocational higher education system within the digital public health sector. Our proposed Blockchain-driven Vocational Higher Education Framework aims to bridge the gap between industry, academia, and research by leveraging the unique capabilities of blockchain technology. The framework comprises two main components: the Blockchain-Enhanced Collaborative Learning Model (BECLM) and the Collaborative Knowledge Exchange Strategy (BECF). These components facilitate seamless collaboration and real-time data sharing among stakeholders, ensuring that educational content is both relevant and current. Our experimental results indicate that the integration of smart contracts, token-based incentives, and advanced data analytics within this framework provides a scalable and trustworthy collaboration infrastructure that supports employability-oriented skill development in the digital public health sector by aligning educational outcomes with industry demands. The framework sets a new benchmark for collaboration, ultimately contributing to the development of a more skilled and adaptable workforce in the digital public health domain. The ICU mortality prediction study serves as a high-stakes digital public health case to demonstrate that the proposed framework can reliably support real-world multi-stakeholder collaboration with privacy constraints, traceable contributions, and incentive-aligned participation, which are also essential requirements for authentic vocational training scenarios.

Despite the promising results, there are two notable limitations to our approach. First, the implementation of blockchain technology in educational settings requires significant initial investment and technical expertise, which may pose a barrier for some institutions. Future research should explore cost-effective solutions and provide guidelines for institutions with limited resources to adopt this framework. Second, while the framework enhances collaboration and data sharing, it also raises concerns about data privacy and security. As blockchain technology evolves, it is crucial to develop robust security protocols and privacy measures to protect sensitive information. Future work should focus on addressing these challenges to ensure the safe and ethical use of blockchain in education. By overcoming these limitations, we anticipate that our framework will play a pivotal role in transforming vocational higher education and fostering a more dynamic and responsive educational ecosystem.

## Data Availability

The original contributions presented in the study are included in the article/supplementary material, further inquiries can be directed to the corresponding author.

## Footnotes

1 MIMIC-III: https://physionet.org/content/mimiciii/1.4/.

2 eICU-CRD: https://physionet.org/content/eicu-crd/2.0/.
